# Are known biomarkers for asthma present in early infancy?

**DOI:** 10.1186/1710-1492-10-S1-A48

**Published:** 2014-03-03

**Authors:** M Ayanna Boyce, Sanja Stanojevic, Krzysztof Kowalik, Susan Balkovec, Felix Ratjen, Malcolm R Sears, Padmaja Subbarao

**Affiliations:** 1 Faculty of Applied Health Sciences, University of Waterloo, Waterloo, Canada; 2 Respiratory Medicine, Hospital for Sick Children, Toronto, Canada; 3 Department of Physiology, McGill University, Montreal, Canada; 4 Department of Medicine, McMaster University, Hamilton, Canada

## Background

Exhaled nitric oxide (FE_NO_) is a biomarker for eosinophilic airway inflammation [1]. Elevated FE_NO_ has been proposed as a marker for diagnosing asthma and predicting asthma exacerbations [2]. We wished to examine the association between known asthma risk factors and FE_NO_. A sub cohort of children (n=222) participating in the Canadian Healthy Infant Longitudinal Development (CHILD) study underwent infant pulmonary function tests (IPFTs) during the first year of life.

## Methods

Risk factors were obtained from a subset of available CHILD questionnaires administered prenatally and 3 times during the first year of life. FE_NO_ was collected using a multiple-breath sampling technique during quiet tidal breathing at the 3 month visit, 1 year visit, or both visits. Prenatal smoke exposure was defined as any maternal smoking, including mothers who stopped or cut down on smoking during pregnancy. Postnatal smoke exposure was defined as any exposure in or away from the home up to 1 year of age. Parental asthma was defined as self-reported or doctor diagnosed asthma. Parental atopic status was confirmed by allergy skin tests. T-tests with Bonferroni correction for multiple comparisons were used to compare FE_NO_ in the exposed and unexposed groups (α=0.004).

## Results

At the 3 month visit, 134 infants attended the IPFT lab, and 84 of 117 attempted eNO tests were successfully analyzed; mean FE_NO_ was 16.8±8.1ppb. At the 1 year visit, 181 infants attended the IPFT lab and 138 of 158 attempted eNO tests were successfully analyzed; mean FE_NO_ was 15.3±9.7ppb. Prenatal smoking rates were low (3% and 6%) and showed no association with FE_NO_ (Table 1). Postnatal smoke exposure was also not associated with FE_NO_. FE_NO_ was not statistically different in infants whose mothers or fathers had a history of asthma or atopic status, compared to those without. Having siblings was not significantly associated with FE_NO_ after applying the Bonferroni correction.

**Table 1:** 

Variable	Testing Time Point		Sample Size	Mean ± SD (ppb)	P value
Prenatal Smoke Exposure	3 months	Yes	2	13.12 ± 0.8	0.072
		No	57	15.40 ± 7.7	
	1 Year	Yes	6	21.12 ± 14.6	0.405
		No	100	15.63 ± 9.5	
Postnatal Smoke Exposure	3 months	Yes	14	15.13 ± 7.5	0.471
		No	64	16.76 ± 7.6	
	1 Year	Yes	38	13.72 ± 8.2	0.233
		No	99	15.73 ± 10.1	
Maternal Atopic Status	3 months	Yes	47	16.72 ± 7.5	0.458
		No	19	15.19 ± 7.5	
	1 Year	Yes	87	15.11 ± 10.0	0.638
		No	40	15.94 ± 8.9	
Maternal Asthma	3 months	Yes	16	17.23 ± 8.2	0.495
		No	49	15.62 ± 7.5	
	1 Year	Yes	29	14.50 ± 7.9	0.460
		No	88	15.86 ± 10.3	
Paternal Atopic Status	3 months	Yes	55	16.32 ± 7.4	0.925
		No	12	16.07 ± 8.4	
	1 Year	Yes	87	15.13 ± 9.4	0.085
		No	21	19.24 ± 9.5	
Paternal Asthma	3 months	Yes	10	15.45 ± 10.0	0.962
		No	48	15.28 ± 7.2	
	1 Year	Yes	20	19.42 ± 10.1	0.081
		No	80	14.89 ± 9.5	
Sibling	3 months	Yes	30	13.54 ± 8.2	0.065
		No	29	17.17 ± 6.5	
	1 Year	Yes	46	13.27 ± 10.0	0.015
		No	60	17.98 ± 9.4	

## Conclusions

Smoke exposure was not related to FE_NO_, however no nicotine biomarker was assessed and smoking rates were low. Maternal and paternal histories were not associated with FE_NO_ levels in healthy children up to 1 year of age. None of the risk factors were statistically significantly associated with FE_NO_, however infants with siblings were observed to have a lower FE_NO_ than infants without siblings at the 1 year visit. A larger sample size is required to increase the power of these tests. Further factors must be studied to explain the variation in FE_NO_ measures seen.
